# Assessing the Accuracy of Diagnostic Capabilities of Large Language Models

**DOI:** 10.3390/diagnostics15131657

**Published:** 2025-06-29

**Authors:** Andrada Elena Urda-Cîmpean, Daniel-Corneliu Leucuța, Cristina Drugan, Alina-Gabriela Duțu, Tudor Călinici, Tudor Drugan

**Affiliations:** 1 Department of Medical Informatics and Biostatistics, Iuliu Hațieganu University of Medicine and Pharmacy, 400349 Cluj-Napoca, Romania; 2 Department of Medical Biochemistry, Iuliu Hațieganu University of Medicine and Pharmacy, 400349 Cluj-Napoca, Romania

**Keywords:** diagnostic accuracy, large language modes, artificial intelligence, medical education

## Abstract

**Background:** In recent years, numerous artificial intelligence applications, especially generative large language models, have evolved in the medical field. This study conducted a structured comparative analysis of four leading generative large language models (LLMs)—ChatGPT-4o (OpenAI), Grok-3 (xAI), Gemini-2.0 Flash (Google), and DeepSeek-V3 (DeepSeek)—to evaluate their diagnostic performance in clinical case scenarios. **Methods:** We assessed medical knowledge recall and clinical reasoning capabilities through staged, progressively complex cases, with responses graded by expert raters using a 0–5 scale. **Results:** All models performed better on knowledge-based questions than on reasoning tasks, highlighting the ongoing limitations in contextual diagnostic synthesis. Overall, DeepSeek outperformed the other models, achieving significantly higher scores across all evaluation dimensions (*p* < 0.05), particularly in regards to medical reasoning tasks. **Conclusions:** While these findings support the feasibility of using LLMs for medical training and decision support, the study emphasizes the need for improved interpretability, prompt optimization, and rigorous benchmarking to ensure clinical reliability. This structured, comparative approach contributes to ongoing efforts to establish standardized evaluation frameworks for integrating LLMs into diagnostic workflows.

## 1. Introduction

Artificial Intelligence (AI) is rapidly reshaping the medical landscape by redefining how information is synthesized, interpreted, and applied across clinical, administrative, and educational domains [1,2]. A significant innovation in AI is the development of large language models (LLMs), transformer-based architectures with strong contextual and semantic capabilities. Despite shared underlying structures, these models excel at handling diverse, multi-modal data, making them well-suited for biomedical and healthcare applications where varied data types are the norm [3]. Unlike traditional rule-based AI, LLMs support multi-turn (conversations that span multiple exchanges or dialogue turns), dynamic interactions that mirror human dialogue [2].

Models such as GPT-4o, Gemini, Grok, and DeepSeek, are trained on massive biomedical and clinical corpora, hold billions of parameters, and already support tasks like triage, chronic disease management, decision support, and patient education. They offer scalable, immediate, and personalized informational support [2,4]. Benchmarking on PromptBench reveals that commercial LLMs, including ChatGPT, Claude, and Gemini, outperform traditional AI systems (like neural translators and symbolic inference engines) in regards to contextual understanding, although they remain limited for formal logic and symbolic reasoning [5].

In medical education, LLMs like ChatGPT are used as pilot approaches in various domains, such as radiotherapy education, helping to demystify complex technical concepts, like dose distribution or radiation side effects [2]. Unlike human standardized patients (SPs), which are expensive and resource-intensive, LLMs can act as a low-cost, highly adaptable standardized patient [6]. Additionally, GPT-4 has demonstrated performance close to that of human evaluators by identifying both factual mistakes and reasoning flaws in student work. By comparison, AI systems using rule-based machine learning showed difficulty interpreting ambiguous language, reinforcing the superior contextual understanding of LLMs [7].

While early results show that LLMs (like ChatGPT) can enhance learner engagement and knowledge retention in medical training, further refinement is needed to ensure reliability, transparency, and clinical trust. Despite these advantages, serious challenges remain. LLMs sometimes “hallucinate”, i.e., they produce verbose or misleading content and lack transparent reasoning, especially in high-stakes contexts [2,3,4]. However, LLM performance in real-world clinical environments still lags behind that of trained physicians. This underperformance is often attributed to issues with interpretability, inconsistent reasoning, the risk of biased or outdated responses, and ethical concerns related to privacy and accountability [2]. While they perform well on simpler tasks, they often falter when it comes to complex, ambiguous clinical problems, particularly when lacking fine-tuned, domain-specific data [8,9]. Even when tested on licensing exams, LLMs showed performance highly dependent on prompt quality and pretraining exposure [10].

Critically, diagnostic reasoning requires more than fact retrieval. Forming a clinical diagnosis involves analyzing patient data, applying medical knowledge, and testing diagnostic hypotheses—a process known as clinical diagnostic reasoning [11].

Recent investigations into LLMs for use in clinical diagnostics shows potential in narrow specialties such as pediatrics [12], pulmonology [13], radiology [14], and even for application to rare disorders [15] or in self-diagnosis contexts [4], demonstrating LLMs’ capacity to interpret complex data and propose credible differential diagnoses, particularly for rare cases. Moreover, comparative studies highlighted that chain-of-thought (CoT) prompts significantly enhanced diagnostic reasoning by structuring inputs in a way that emulates expert thinking, whereas standard prompts often produced overly verbose outputs that hinder decision-making effectiveness [16]. In clinical decision-making contexts in fields such as oncology and cardiology, LLMs produced richer and more personalized treatment suggestions than do database-driven tools, although they occasionally proposed unverified or hallucinated interventions, highlighting the need for clinical oversight [17].

Attempts to enhance LLMs through knowledge graphs have shown promise, particularly in zero-shot settings (no prior examples given to the LLMs), although limitations remain. A notable limitation lies in their reduced capacity to accurately capture all clinically relevant concepts, especially those that are indirect, nuanced, or context-dependent, yet critical for comprehensive diagnostic reasoning [11]. Their performance, particularly in complex or multilingual clinical environments, remains uneven—LLMs excel in English, but show gaps in low-resource languages [18]. Studies on post-operative patient support indicate that LLMs, while occasionally imprecise, offer superior clarity, empathy, and conversational quality compared to the results for rigid rule-based assistants—suggesting that a hybrid approach may offer the best of both systems [19].

Importantly, real-world clinical scenarios are inherently complex and surpass multiple-choice medical examination. Therefore, a comprehensive evaluation of LLMs must consider not only their ability to generate medically accurate language, but also their capacity to retrieve applied knowledge to context-specific problems and to perform diagnostic reasoning [20].

Despite growing interest, a systematic approach to assessing diagnostic reasoning across multiple LLMs is still lacking. Most studies have not provided consistent or scalable scoring frameworks to capture diagnostic capabilities. The current tendency is to evaluate responses with metrics based on n-gram overlap (like BLEU or ROUGE), or to count the attempts until the “correct” diagnosis was delivered. Such approaches oversimplify the multifactorial nature of clinical decision making [21,22].

The use of LLMs for diagnosis and self-diagnosis has already become a reality in medical practice, and the LLM capabilities have been evolving at a very rapid pace. In light of the emerging interactions between physicians and AI, it has become necessary to assess the ability of current systems to appropriately respond to accurately presented medical cases. In this context, our study aimed to conduct a comparative analysis of four LLMs (in their free versions) by presenting them with a series of clinical cases and evaluating their responses, as if they had been provided by medical professionals.

## 2. Materials and Methods

**Figure 1:**
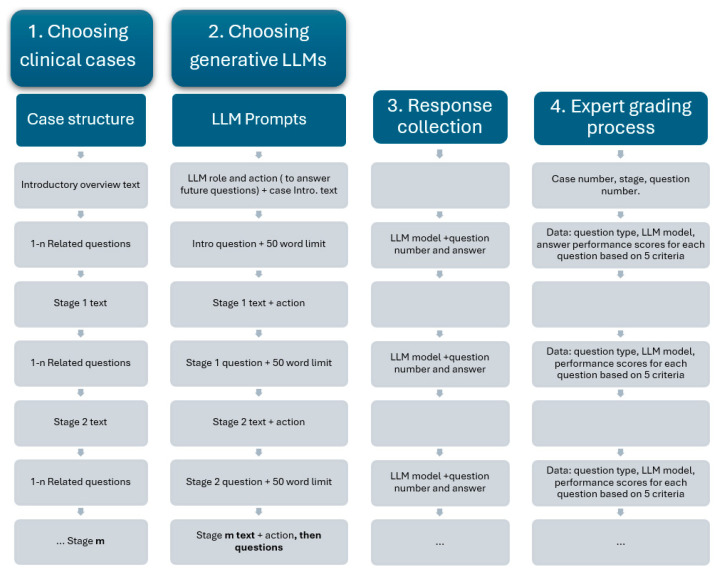
Flowchart of the methodological steps used in the study.

LLM assessment was achieved using complex medical case presentations, followed by structured questions designed to assess two key capabilities: medical knowledge recall (such as identifying likely diagnoses and interpreting test results) and clinical reasoning capabilities (giving reasons for diagnosis choices and proposing the next steps in patient management). The methodological steps are presented in Figure 1.

The clinical cases used in this study were randomly selected from our university’s internal case database, which is routinely used in problem-based learning (PBL) for medical students. Each case was inspired by real clinical encounters and then carefully adapted by experienced medical educators to maximize educational value. These cases were structured using a standardized, staged format, where clinical information was disclosed progressively to simulate real-time diagnostic reasoning. After each stage, a series of questions specifically related to the newly introduced information was posed, assessing the student’s ability to integrate and apply context-specific data. Cases were selected based on their focus on diagnostic pathways, including primary diagnoses, complications, and adverse reactions, and were classified by difficulty level and medical specialty. This incremental and structured approach ensured consistency in our study, while enabling meaningful evaluation of both medical knowledge recall and applied clinical reasoning across different LLMs.

**Figure 2:**
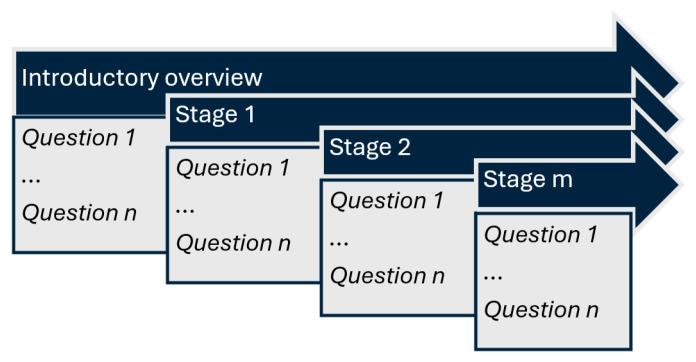
Conceptual clinical case stage/context complexity and structure.

The available data included the patient’s current issue and symptoms, general context, medical history, laboratory results, medical imaging results, and follow-up results. Each case comprised an introductory overview and a staged disclosure (additional 6 to 10 contextual elements, gradually presented). At each stage, both the understanding of essential medical concepts and the ability to apply them were assessed through progressive questioning (4 to 10 questions). The complexity of each case increased progressively, as contextual data was incrementally added along with more clinical information (Figure 2).

For the AI assessment, the following generative LLMs were chosen: the Chat GPT 4o model (OpenAI, San Francisco, CA, USA), the Grok 3 model (xAI, San Francisco, CA, USA), the Gemini 2.0 flash model (Google DeepMind, London, UK), and the DeepSeek V3 model (DeepSeek, Hangzhou, China), using their free public interfaces. To ensure consistent responses, each model received identical prompts in English. The LLMs’ responses to the questions at each stage were expected to integrate all information provided up to that point, including both general and stage-specific contextual data. Specifically, the application-oriented questions required reference not only to the most recently introduced context, but also to the cumulative contextual information presented throughout the case.

This approach was chosen to evaluate the responses of LLMs in several potential use-case scenarios: first, a physician verifying the accuracy of their clinical reasoning by presenting the case to the AI system; second, a patient assessing whether their case has been appropriately managed by the medical team; and third, a potential future scenario in which AI systems may independently perform complex diagnostic reasoning.

### 2.1. Prompting Strategy

For the discussion of medical cases, each of the LLMs was asked to assume the role of a medical student, analyze the given clinical scenario, and respond to several questions. Next, the introductory overview was provided. Questions were delivered one at a time, with responses capped at 50 words. New contextual data were incrementally introduced, followed by related questions, with responses capped at 50 words. This strategy was applied repeatedly, according to the number of stages specific to each case. Each question was pre-classified as either a test of subject-specific medical knowledge or a test of the ability to apply that knowledge in medical reasoning.

### 2.2. Assessment of LLMs Responses

Two medical expert evaluators (experienced in problem-based learning and previously involved in using such clinical cases with medical students), who were blinded to the source of each set of answers, independently scored the responses on a 0–5 scale. For each question, the LLM response was evaluated and the performance scores were awarded based on the following criteria:

1. Question comprehension—the ability to correctly interpret the clinical query’s intent and scope;
2. Medical knowledge on the subject—the depth and accuracy of factual medical information presented by LLMs;
3. Understanding the medical context—the ability to appropriately apply medical knowledge to the specific case details;
4. Correctness—the accuracy of the final diagnosis and management recommendations;
5. Clarity in formulating the answer—organization, coherence, and readability of the response.

### 2.3. Data Analysis

Descriptive statistics for qualitative data were reported as counts and/or percentages. Given the ordinal nature of the scoring system (0–5 scale), non-parametric statistical methods were employed. Although the score distributions were skewed toward higher values—indicating generally strong LLM performance—means and standard deviation intervals (±SD) were also computed to enhance interpretability in the graphical representations.

Overall differences in LLM performance scores were assessed using the Kruskal–Wallis test. For post hoc pairwise comparisons, the Dwass–Steel–Critchlow–Fligner method was applied. Statistical significance was defined as *p* < 0.05.

Data analysis was performed using Microsoft Excel (Office Professional Plus 2021) and the open-source statistical software Jamovi (version 2.3.28) based on R language [23,24].

## 3. Results

**Figure 3:**
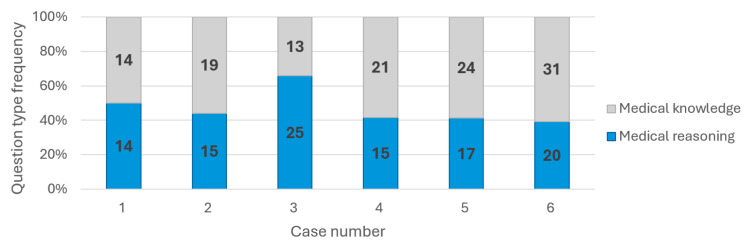
Question type distribution for each clinical case.

A total of six complex medical cases were selected, encompassing a range of specialties, patient trajectories, and comorbidities. Each case included a series of questions designed to assess both medical knowledge (122 questions in total) and clinical reasoning capabilities (106 questions in total) (Figure 3). The number of questions per case varied between 28 and 51.

All 231 questions were presented in sequence, along with their corresponding clinical contexts, to the four LLMs described in the Materials and Methods section. The responses received were evaluated by two medical experts, who established a consensus score for each individual answer. Overall, all LLM systems received high scores for their responses, with the proportion of scores of 4 or 5 (good or very good responses) exceeding 80%.

Due to the scoring system and the distribution of scores being skewed toward the maximum, the data did not follow a normal distribution. The Kolmogorov–Smirnov test yielded a *p*-value of less than 0.001. For the comparison of LLMs, a non-parametric statistical test was used; however, the graphical representation employed mean and standard deviation for better interpretability, as box plots were affected by the ceiling effect in score distribution.

The comparative analysis followed three main directions: an overall comparison of the scores obtained, a comparison of scores for medical knowledge questions, and a comparison of scores for clinical reasoning questions among the four LLMs.

### 3.1. Overall Comparison

**Figure 4:**
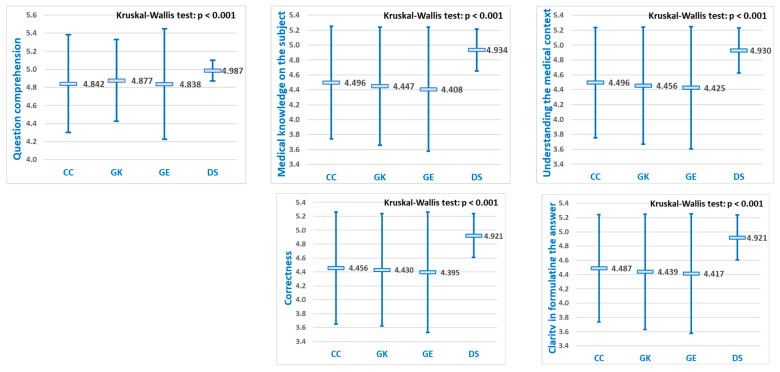
LLMs comparison of mean performance scores (±SD) according to the five criteria used to assess diagnostic capabilities (CG = Chat GPT, GK = Grok, GE = Gemini, and DS = DeepSeek).

All LLMs scores assigned by experts were compared using a Kruskal–Wallis test, according to each of the five criteria for response evaluation, to establish whether there was any statistically significant difference in performance scores (Figure 4):

Since in all five criteria assessing medical reasoning, statistically significant differences were found according to the Kruskal–Wallis test (*p* < 0.001), pairwise comparisons were made using the Dwass–Steel–Critchlow–Fligner method (Table 1).

Across all five evaluation criteria, DeepSeek consistently outperformed each of the other models—ChatGPT, Grok, and Gemini—with statistically significant differences in every comparison. As shown in Figure 4, DeepSeek achieved the highest overall scores in each case.

### 3.2. Comparison for Medical Knowledge

**Figure 5:**
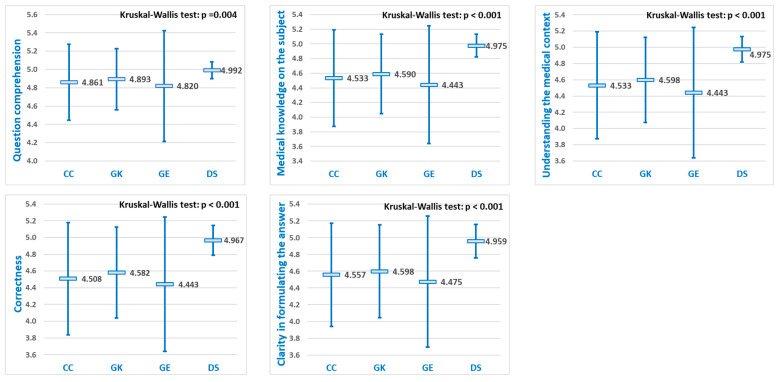
LLMs comparison of mean performance scores (±SD) for questions ***regarding medical knowledge***, according to the five criteria (CG = Chat GPT, GK = Grok, GE = Gemini, and DS = DeepSeek).

The LLMs’ response data was then split according to each type of question. To comprehensively assess diagnostic capabilities, the mean performance score for responses to questions regarding medical knowledge was computed according to the five criteria for each LLM, and the results are represented graphically (Figure 5).

Given the statistically significant differences observed across all five key dimensions of diagnostic capabilities for questions regarding ***medical knowledge questions*** *(Kruskal*–*Wallis test p < 0.001)*, pairwise comparisons were performed using the Dwass–Steel–Critchlow–Fligner method (Table 2).

When focusing on questions related to ***medical knowledge***, DeepSeek scored significantly higher than ChatGPT, Grok, and Gemini across all five evaluation criteria. As illustrated in Figure 5 DeepSeek consistently achieved the highest scores in each comparison, highlighting its superior performance in this area.

### 3.3. Comparison for Medical Reasoning

**Figure 6:**
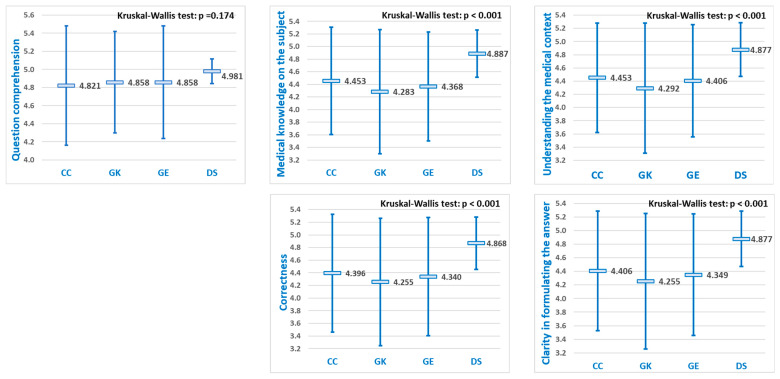
LLMs comparison of mean performance scores (±SD) for questions regarding ***medical reasoning***, according to the five criteria (CG = Chat GPT, GK = Grok, GE = Gemini, and DS = DeepSeek).

Next, to assess diagnostic capabilities of LLMs, the mean performance score for responses to clinical reasoning questions was calculated based on the five criteria for each LLM, and the results are represented graphically (Figure 6).

Given the statistically significant differences observed across all five key dimensions of diagnostic capabilities regarding ***medical reasoning questions*** (Kruskal–Wallis test, *p* < 0.001), pairwise comparisons were conducted using the Dwass–Steel–Critchlow–Fligner method (Table 3).

When focusing on questions related to medical reasoning, DeepSeek scored significantly higher than ChatGPT, Grok, and Gemini across only the last four evaluation criteria. As illustrated in Figure 6, DeepSeek consistently achieved the highest scores in each comparison, highlighting its superior performance in this area.

## 4. Discussion

All four LLMs were chosen based on their user popularity and performance. The six randomly chosen cases varied in pathology and medical complexity, while their related questions evaluated both medical knowledge and reasoning capabilities in an almost similar manner (Figure 3). We conducted a comparative analysis of four leading generative LLMs, and the following key observations highlighted their performance in diagnosing medical cases.

For each of the five diagnostic evaluation criteria, significant differences were observed in the mean performance scores of the LLMs (Figure 4). Pairwise comparisons revealed that DeepSeek achieved statistically significantly higher scores than the other three LLMs (*p* < 0.05) for all five evaluation criteria, while no significant differences were observed among ChatGPT, Grok, and Gemini (Table 3).

Each LLM demonstrated higher mean scores for medical knowledge questions compared to medical reasoning questions (Figure 5 and Figure 6). This outcome was anticipated, as retrieving factual information is generally less complex for software models to perform than is applying knowledge to clinical scenarios.

Focusing solely on the ***medical knowledge questions***, significant differences in mean performance scores were observed among the LLMs, for each of the five evaluation criteria (Figure 5). Among these criteria, “*Question comprehention*” consistently yielded the highest mean scores for each of the four LLMs. Pairwise comparisons indicated that DeepSeek achieved significantly higher scores than the other three LLMs (*p* < 0.05) across all five evaluation criteria, whereas no significant differences were found among ChatGPT, Grok, and Gemini (Table 2).

Focusing specifically on ***medical reasoning questions***, “*Question comprehention*” consistently produced the highest mean scores for all four LLMs, without statistically significant differences between them. In contrast, for the remaining four evaluation criteria, significant differences in mean performance scores were found (Figure 6). Pairwise comparisons indicated that DeepSeek achieved significantly higher scores than the other three LLMs (*p* < 0.05) across the remaining four evaluation criteria, whereas no significant differences were found among ChatGPT, Grok, and Gemini (Table 3).

Assessing the diagnostic performance of large language models (LLMs) across diverse clinical scenarios has so far been approached using a range of evaluation metrics, including precision, accuracy, recall, and F1-score, to quantify diagnostic effectiveness. Additionally, in some studies, both qualitative and quantitative approaches were used to analyze LLM outputs—qualitative assessments focused on response quality, consistency, and structure, while quantitative metrics, such as top-1, top-3, and top-5 accuracy, evaluated the model’s ability to generate correct diagnoses within its leading predictions [22]. According to Shan G et al., between 2023–2025, many studies evaluated the diagnostic accuracy of several LLMs and concluded that it varied from 25% to 97.8% when compared to that of medical professionals, with a high risk of bias in the majority of studies [25]. On the other hand, our study focused on the capacity of LLMs to answer correctly, in order to check whether they could be safely used as AI assistants in student medical training on diagnostic reasoning. Our analysis showed mean scores starting from 4.25 up to 4.99 (on a 0–5 scale), which we consider good performances for all LLMs, with a better performance for DeepSeek.

The differences observed between LLMs are both objective and potentially subjective. Lower scores were awarded when key factors in case evaluation were overlooked, with systems often being drawn to the first abnormalities presented in the context. This behavior is possibly a consequence of the learning paradigm adopted by the LLMs.

Subjectively, we observed consistent stylistic differences among the models, despite the fact that no prompting directives were provided by the research team. ChatGPT, Grok, and Gemini generally produced narrative-style responses, whereas DeepSeek adopted a more structured format, typically beginning with a brief introductory statement, followed by bullet-pointed subcomponents. Our prompts did not specify whether the expected response should focus on medical knowledge or clinical reasoning, yet DeepSeek was the only LLM that consistently responded to both knowledge-based and reasoning questions, not only with the expected knowledge but also by explicitly connecting the information to the specific case data. In this regard, DeepSeek adhered to the initial instruction in the context-setting prompt to answer within that context, whereas the other systems tended to ignore it, unless the questions explicitly referenced content from the given context. DeepSeek’s superior performance may be attributed to its consistent formatting, stronger prompt adherence, and better contextual integration; further qualitative analysis of response quality is warranted in future research.

As Rider et al. demonstrated through a structured evaluation of six state-of-the-art models using 25 real-world primary immunodeficiency cases, LLMs vary substantially in regards to both diagnostic accuracy and clinical reasoning. While models like GPT-4o reached diagnostic accuracies as high as 96.2%, even the top-performing systems occasionally produced incorrect information or failed to integrate context effectively. These observations underscore the need for more robust evaluation frameworks that assess diagnostic competence beyond simple correctness metrics [15].

Recent progress in large language models (LLMs) has shown that they can help with medical diagnoses, but using structured reasoning methods like chain-of-thought (CoT) is still not common [16]. Some researchers like Liu et al. have used expert-guided CoT in kidney disease diagnosis, but it required training the model specifically for that area. This study takes a different approach by using CoT without extra training (zero-shot approach), which makes the model think more like a doctor and explain its reasoning more clearly [26]. Unlike other studies that focused on tasks like coding for sepsis or summarizing clinical notes, this method directly connects key medical signs—such as high blood pressure or kidney structure description—to possible diagnoses. This better matches how doctors actually work and makes the model’s decisions easier to understand and more useful in real-life situations [16].

When comparing our results with those of Hager P et al., we observed a substantial increase in diagnostic accuracy and response capability in our study. This improvement can be attributed to the use of prefabricated scenarios with high-quality educational context, as opposed to the presentation of real cases, which may include variability and potentially incomplete descriptions [27]. Moreover, the evaluation criteria used in Hager P et al. were extremely rigid, in contrast to the manual interpretation of responses based on five criteria, as implemented in our study [27]. Additionally, our evaluation was performed on numerous successive responses for each case, rather than relying on a single final diagnosis.

Some studies analyzed the use of LLMs specifically in medical education. These models can support patient triage, clinical decision making, and knowledge assessment; however, the accuracy does not always ensure the correct resolution of clinical cases [28]. These findings align with the same potential developmental trajectory as our present research—namely, the integration of LLMs into the training of future physicians—and they similarly conduct a response-level analysis of diagnostic interaction with LLMs [28]. It is also worth highlighting, in this same direction, the approach of Brügge et al., who have taken the next step in the use of LLMs for medical training by attempting to integrate ChatGPT into the education of future medical students [29]—an approach that our work will likely follow in the near future.

Future research should adopt comprehensive, multi-dimensional evaluation tools that capture not only the accuracy, but also the coherence, justification, and adaptability of reasoning. Additionally, systematic comparisons across multiple LLMs using real medical data are essential to gather information on their safe, ethical, and effective deployment in healthcare settings.

### 4.1. Strengths

Regarding the strengths of this study, we would first like to emphasize that the use of highly standardized educational cases—lacking the inherent variability of real-world scenarios—was an important choice for assessing the diagnostic capabilities of LLMs that were not trained on real clinical data. Second, the use of public LLM interfaces made our study easily reproducible and verifiable. Third and finally, the complexity of the cases employed—comprising a total of 228 questions applied across 4 LLMs—generated over 1000 responses, each of which was scored using five criteria, thereby enhancing the study’s statistical analysis power and enabling a robust comparison of the results.

### 4.2. Limitations

Regarding the limitations, we aim to highlight the most significant ones. First, the evaluation was conducted using only six complex clinical cases which, although diverse in terms of pathology and structure, may not fully capture the breadth of real-world clinical scenarios. Second, all cases were drawn from a single institutional database, which may introduce potential biases in medical decision-making approaches. Third, the cases were educational in nature and specifically designed to provide all necessary information; due to their idealized format, they are somewhat removed from real-world clinical complexity.

The testing environment, designed with a structured, staged disclosure of information and a 50-word limit on responses, does not fully replicate how generative language models are typically used in real-world settings, where longer and more dynamic interactions are possible. While this format ensured consistency, it may have constrained the depth of model reasoning and response formulation. In addition, differences in model architecture may have led to varied interpretations of the “medical student” prompt. Future work should explore prompt refinement and evaluate responses across different clinical roles (e.g., patient, consultant) for greater consistency.

Although each model was prompted identically, prompting itself remains a source of potential bias, as different LLM architectures may interpret the same instructions differently. The use of the “medical student” role may not standardize behavior across models, limiting the comparability of their performance.

The evaluation of responses by two medical experts with experience in problem-based learning could also be a source of error, as subjective scoring may have influenced the results.

Finally, the findings reflect the performance of specific LLM versions (GPT-4o, Grok 3, Gemini 2.0 Flash, and DeepSeek V3) at the time of the study. Future updates or changes to model architectures may lead to different outcomes, affecting the generalizability and reproducibility of our findings.

## 5. Conclusions

The comparative analysis of the evaluated LLMs demonstrated that current large language models are capable of generating highly accurate diagnostic responses when presented with well-structured, educationally optimized clinical cases. Among the four models tested, DeepSeek consistently outperformed the others, achieving significantly higher scores across all evaluation criteria. It was particularly distinguished by its superior performance on questions assessing both medical knowledge and applied clinical medical reasoning, although the greatest differences were observed in regards to knowledge-based tasks. While GPT-4o, Grok, and Gemini showed comparable performance levels, DeepSeek’s accuracy and contextual integration set it apart, suggesting its greater potential for supporting medical training and diagnostic decision making in controlled educational settings.

## Figures and Tables

**Table 1:** Pairwise comparisons of LLMs performance scores according to the five criteria to assess diagnostic capabilities.

LLMs Type	Understanding the Question *p*-Value *	Medical Knowledge of the Subject *p*-Value *	Understanding the Medical Context *p*-Value *	Correctness *p*-Value *	Clarity in Formulating the Answer *p*-Value *
CC-DS	**<0.001**	**<0.001**	**<0.001**	**<0.001**	**<0.001**
CC-GE	0.991	0.688	0.858	0.893	0.872
CC-GK	0.955	0.923	0.973	0.973	0.959
DS-GE	**<0.001**	**<0.001**	**<0.001**	**<0.001**	**<0.001**
DS-GK	**0.002**	**<0.001**	**<0.001**	**<0.001**	**<0.001**
GE-GK	0.996	0.965	0.984	0.992	0.994

* Pairwise comparisons, bold values were statistically significant; CG = Chat GPT, GK = Grok, GE = Gemini, and DS = DeepSeek.

**Table 2:** Pairwise comparisons of LLMs performance scores for questions regarding ***medical knowledge***, according to the five criteria to assess diagnostic capabilities.

LLMs Type	Understanding the Question *p*-Value *	Medical Knowledge of the Subject *p*-Value *	Understanding the Medical Context *p*-Value *	Correctness *p*-Value *	Clarity in Formulating the Answer *p*-Value *
CC-DS	**0.003**	**<0.001**	**<0.001**	**<0.001**	**<0.001**
CC-GE	0.996	0.893	0.893	0.975	0.945
CC-GK	0.969	0.987	0.981	0.957	0.985
DS-GE	**0.002**	**<0.001**	**<0.001**	**<0.001**	**<0.001**
DS-GK	**0.009**	**<0.001**	**<0.001**	**<0.001**	**<0.001**
GE-GK	0.91	0.716	0.683	0.785	0.8

* Pairwise comparisons, bold values were statistically significant; CG = Chat GPT, GK = Grok, GE = Gemini, and DS = DeepSeek.

**Table 3:** Pairwise comparisons of LLMs performance scores for questions regarding ***medical reasoning***, according to the five criteria to assess diagnostic capabilities.

LLMs Type	Understanding the Question *p*-Value *	Medical Knowledge of the Subject *p*-Value *	Understanding the Medical Context *p*-Value *	Correctness *p*-Value *	Clarity in Formulating the Answer *p*-Value *
CC-DS	0.124	**<0.001**	**<0.001**	**<0.001**	**<0.001**
CC-GE	0.868	0.838	0.978	0.942	0.957
CC-GK	0.993	0.617	0.747	0.695	0.721
DS-GE	0.455	**<0.001**	**<0.001**	**<0.001**	**<0.001**
DS-GK	0.202	**<0.001**	**<0.001**	**<0.001**	**<0.001**
GE-GK	0.957	0.977	0.926	0.95	0.946

* Pairwise comparisons, bold values were statistically significant; CG = Chat GPT, GK = Grok, GE = Gemini, and DS = DeepSeek.

## Data Availability

The datasets presented in this article are not readily available because the data supporting the findings of this study are not publicly available due to institutional restrictions. The dataset used is the property of Iuliu Hațieganu University of Medicine and Pharmacy, and access is governed by internal policies and ethical guidelines.
